# Supplementary figures and images for: Tracing the evolution of key traits in dorid nudibranchs
Source: PLoS One. 2025 Apr 2;20(4):e0317704. doi: 10.1371/journal.pone.0317704 (PMC11964261; doi:10.1371/journal.pone.0317704)

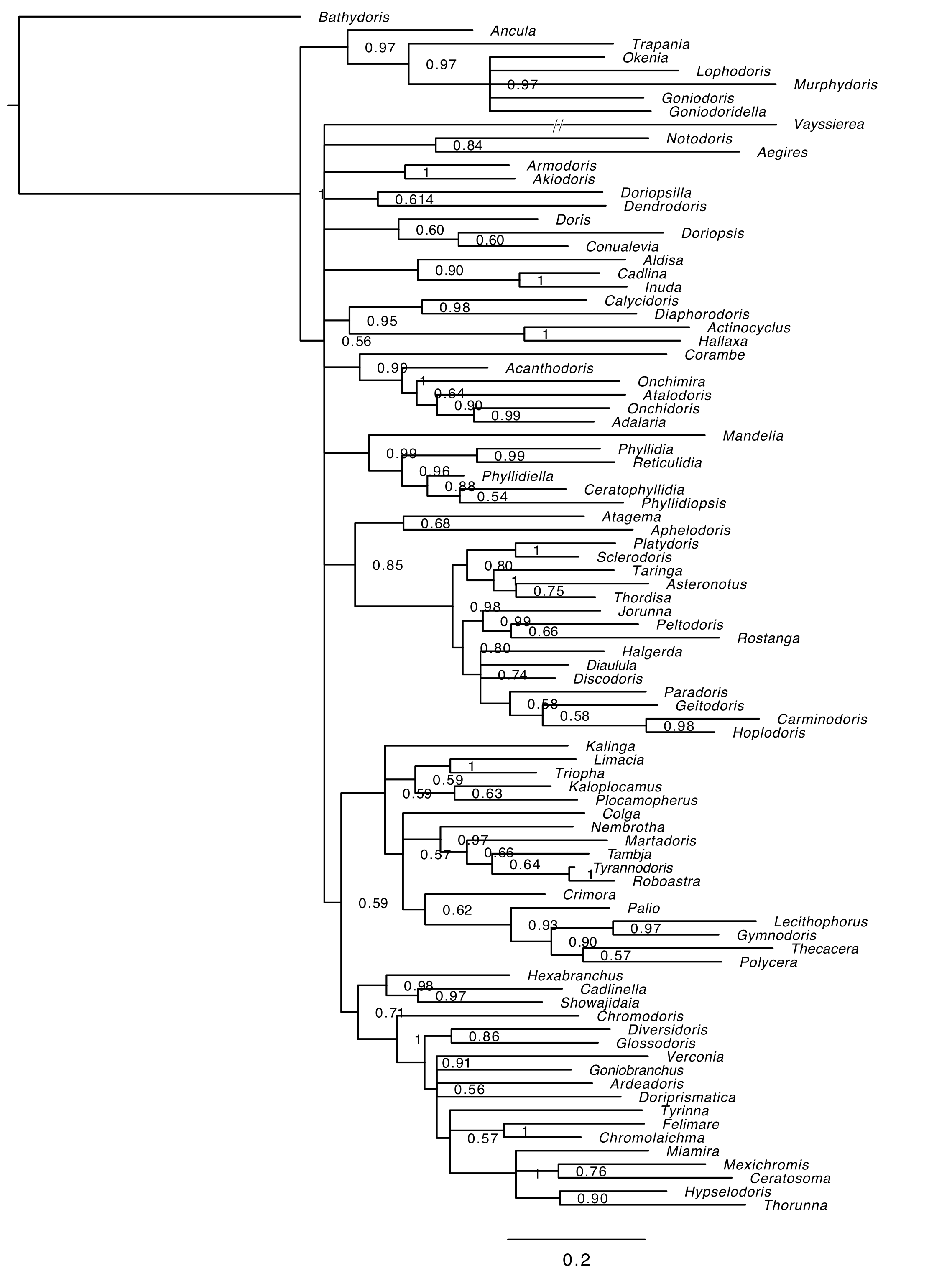

Supplement: S1 Fig — (TIF) [file pone.0317704.s001.tif]
