# Supplementary material for: Tracing the evolution of key traits in dorid nudibranchs
Source: PLoS One. 2025 Apr 2;20(4):e0317704. doi: 10.1371/journal.pone.0317704 (PMC11964261; doi:10.1371/journal.pone.0317704)
Supplement: S1 File — (DOCX) [file pone.0317704.s002.docx]

**Supplementary references (Table 1)**

1. Johnson, S., 2006 (Feb 1) *Actinocyclus verrucosus* on prey sponge. *[Message in] Sea Slug Forum.* Australian Museum, Sydney. Available from http://www.seaslugforum.net/find/15680

2. [McDonald, G. R. & Nybakken, J. W. List of the worldwide food habits of nudibranchs. Available at https://escholarship.org/uc/item/0g75h1q3 (1997).](http://paperpile.com/b/RfW36U/jStB)

3. [Nimbs, M. J. & Smith, S. D. A. An illustrated inventory of the sea slugs of New South Wales, Australia (Gastropoda: Heterobranchia). *Proc. R. Soc. Vic.* **128**, 44 (2016).](http://paperpile.com/b/RfW36U/X1Lc)

4. [Goddard, J. H. Berthella (Opisthobranchia: Pleurobranchidae) from the northeast Pacific Ocean prey on plakinid sponges (Homoscleromorpha: Plakinidae). *The Veliger* **49**, 97–100 (2007).](http://paperpile.com/b/RfW36U/Ksae)

5. [Nimbs, M. J., Hutton, I., Davis, T. R., Larkin, M. F. & Smith, S. D. A. The heterobranch sea slugs of Lord Howe Island, NSW, Australia (Mollusca: Gastropoda). *Proc. R. Soc. Vic.* **132**, 12 (2020).](http://paperpile.com/b/RfW36U/JzeT)

6. [Templado, J., Luque, Á. & Ortea, J. A new species of *Aegines* Lovén, 1844 (Opisthobranchia: Doridacea: Aegiretidae) from the Caribean sea: *Aegines ortizi* spec. nov., with comparative descriptions of the North Atlantic species of this genus. *Veliger* **29**, 303–307 (1987).](http://paperpile.com/b/RfW36U/hJB9)

7. Rudman, W.B., 2000 (January 19) *Aegires villosus* Farran, 1905. *[In] Sea Slug Forum.* Australian Museum, Sydney. Available from http://www.seaslugforum.net/find/aegivill

8. [Picton, B. E. & Christine, C. M. Nudibranchs of the British Isles : Food preferences.](http://paperpile.com/b/RfW36U/OYZf) <http://www.seaslug.org.uk/nudibranchs/foodpref.html> [(1994).](http://paperpile.com/b/RfW36U/OYZf)

9. [Fahey, S. J. & Carroll, A. R. Natural products isolated from species of *Halgerda bergh*, 1880 (Mollusca: Nudibranchia) and their ecological and evolutionary implications. *J. Chem. Ecol.* **33**, 1226–1234 (2007).](http://paperpile.com/b/RfW36U/IaU2)

10. [Dean, L. J. & Prinsep, M. R. The chemistry and chemical ecology of nudibranchs. *Nat. Prod. Rep.* **34**, 1359–1390 (2017).](http://paperpile.com/b/RfW36U/Fl60)

11. Rudman, W.B., 2003 (July 11) *Notodoris minor* Eliot, 1904. *[In] Sea Slug Forum.* Australian Museum, Sydney. Available from http://www.seaslugforum.net/find/notomino

12. Alvi, K. A., Peters, B. M., Lisa, H. M. & Phillip, C. 2-Aminoimidazoles and their zinc complexes from Indo-Pacific *Leucetta* sponges and *Notodoris* nudibranchs. [*Tetrahedron* **49**, 329–336 (1993).](http://paperpile.com/b/RfW36U/QWRl)

13. Alvi, K. A., Crews, P. & Loughhead, D. G. Structures and total synthesis of 2-aminoimidazoles from a *Notodoris* nudibranch. [*J. Nat. Prod.* **54**, 1509–1515 (1991).](http://paperpile.com/b/RfW36U/zo6Y)

14. Millen, S. V. & Martynov, A. Redescriptions of the nudibranch genera *Akiodoris* Bergh, 1879 and *Armodoris* Minichev, 1972 (Suborder Doridacea), with a new species of *Akiodoris* and a new family Akiodorididae. [*Proc. Calif, Acad. Sci* **56** (1): 1-22 (2005).](http://paperpile.com/b/RfW36U/tgRZ)

15. [Mollo, E., Gavagnin, M., Carbone, M., Guo, Y.-W. & Cimino, G. Chemical studies on Indo-Pacific *Ceratosoma* nudibranchs illuminate the protective role of their dorsal horn. *Chemoecology* **15**, 31–36 (2005).](http://paperpile.com/b/RfW36U/T4DS)

16. Korshunova, T. [*et al.*](http://paperpile.com/b/RfW36U/gSTz) The Emperor’s *Cadlina*, hidden diversity and gill cavity evolution: new insights for the taxonomy and phylogeny of dorid nudibranchs (Mollusca: Gastropoda). [*Zool. J. Linn. Soc.* **189**, 762–827 (2020).](http://paperpile.com/b/RfW36U/gSTz)

17. Rudman, W.B., 2006 (Jun 29). Comment on *Cadlina laevis* from France by Wilfried Bay-Nouailhat. *[Message in] Sea Slug Forum.* Australian Museum, Sydney. Available from http://www.seaslugforum.net/find/16923

18. [Belmonte, T., Alvim, J., Padula, V. & Muricy, G. Spongivory by nudibranchs on the coast of Rio de Janeiro state, southeastern Brazil. *Spixiana* **38**, 187–195 (2015).](http://paperpile.com/b/RfW36U/VdrA)

19. [Faulkner, D. J. & Ghiselin, M. T. Chemical defense and evolutionary ecology of dorid nudibranchs and some other opisthobranch gastropods.](http://paperpile.com/b/RfW36U/nMA0) *Mar. Ecol. Prog. Ser. Oldendorf* [**13**, 295–301 (1983).](http://paperpile.com/b/RfW36U/nMA0)

20. Valdés, Á. & Campillo, O. A. Redescription and reassessment of [*Cadlina luarna* (Ev. Marcus and Er. Marcus, 1967), comb. nov. (Mollusca, Opisthobranchia, Doridina). *Proc. Natl. Acad. Sci. U. S. A.* **110**, 14815–14816 (2013).](http://paperpile.com/b/RfW36U/pPcg)

21. [Furfaro, G. & Mariottini, P. Check-list of the Nudibranchs (Mollusca Gastropoda) from the biodiversity hot spot ‘Scoglio del Corallo’ (Argentario promontory, Tuscany).](http://paperpile.com/b/RfW36U/PeXL) *Biodiv. J.* [**7**, 67–78 (2016).](http://paperpile.com/b/RfW36U/PeXL)

22. [Cimino, G., De Rosa, S., De Stefano, S., Sodano, G. & Villani, G. Dorid nudibranch elaborates its own chemical defense. *Science* **219**, 1237–1238 (1983).](http://paperpile.com/b/RfW36U/epOT)

23. Deuss, M., 2010 (Mar 11) *Glossodoris cf. symmetricus* feeding in Mayotte Island. *[Message in] Sea Slug Forum.* Australian Museum, Sydney. Available from http://www.seaslugforum.net/find/23322

24. White, A. M. [*et al.* Oxygenated Diterpenes from the Indo-Pacific Nudibranchs *Goniobranchus splendidus* and *Ardeadoris egretta*. *Nat. Prod. Commun.* **11**, 921–924 (2016).](http://paperpile.com/b/RfW36U/FJFR)

25. [Fisch, K. M. *et al.* The potential of Indonesian heterobranchs found around Bunaken Island for the production of bioactive compounds. *Mar. Drugs* **15**, 384 (2017).](http://paperpile.com/b/RfW36U/t7k3)

26. [Ksebati, M. B. & Schmitz, F. J. Sesquiterpene furans and thiosesquiterpenes from the nudibranch *Ceratosoma brevicaudatum*. *J. Nat. Prod.* **51**, 857–861 (1988).](http://paperpile.com/b/RfW36U/YydX)

27. [Magnificent Chromodoris (*Chromodoris magnifica*). *iNaturalist*](http://paperpile.com/b/RfW36U/rROo) <https://www.inaturalist.org/taxa/121048-Chromodoris-magnifica>[.](http://paperpile.com/b/RfW36U/rROo)

28. [Bornancin, L., Bonnard, I., Mills, S. C. & Banaigs, B. Chemical mediation as a structuring element in marine gastropod predator-prey interactions. *Nat. Prod. Rep.* **34**, 644–676 (2017).](http://paperpile.com/b/RfW36U/2e3G)

29. Fontana, A., Mollo, E., Ortea, J., Gavagnin, M. & Cimino, G. Scalarane and homoscalarane compounds from the nudibranchs *Glossodoris sedna* and *Glossodoris dalli*: chemical and biological properties. [*J. Nat. Prod.* **63**, 527–530 (2000).](http://paperpile.com/b/RfW36U/4o0j)

30. Lee, K. K. Chemical investigation of four marine invertebrates [Master’s dissertation, University of Hawai’i at Manoa] [(1991).](http://paperpile.com/b/RfW36U/e1H9)

31. Hertzer, C. [*et al.* Antibacterial scalarane from *Doriprismatica stellata* nudibranchs (Gastropoda, Nudibranchia), egg ribbons, and their dietary sponge *Spongia cf. agaricina* (Demospongiae, Dictyoceratida). *Beilstein J. Org. Chem.* **16**, 1596–1605 (2020).](http://paperpile.com/b/RfW36U/Hhrh)

32. Rudman, W.B., 2000 (June 1) *Hypselodoris picta* (Schultz, 1836). *[In] Sea Slug Forum.* Australian Museum, Sydney. Available from http://www.seaslugforum.net/factsheet/hypspict

33. [Winters, A. E. *et al.* Distribution of defensive metabolites in nudibranch molluscs. *J. Chem. Ecol.* **44**, 384–396 (2018).](http://paperpile.com/b/RfW36U/qHam)

34. Belmonte, T., Sudatti, D., Pereira, R. & Hajdu, G. L. The behavior of nudibranchs (Mollusca, Gastropoda) in relation to secondary metabolites from marine sponges [Poster, Universidade do Estado do Rio de Janeiro and Universidade Federal Fluminense]. Available at https://www.researchgate.net/profile/Renato-Pereira-12/publication/266157287_The_behavior_of_nudibranchs_Mollusca_Gastropoda_in_relation_to_secondary_metabolites_from_marine_sponges/links/54be62640cf218da9391e88d/The-behavior-of-nudibranchs-Mollusca-Gastropoda-in-relation-to-secondary-metabolites-from-marine-sponges.pdf

35. McCuller, M. I. The influence of abiotic and biotic factors on two nudibranchs feeding upon *Membranipora membranacea* in the southern Gulf of Maine [Master’s thesis, University of New Hampshire] (2012).

36. Okuda, R. K. Chemical ecology of some opisthobranch mollusks [Doctoral dissertation, [University of Hawai'i at Manoa] (1983).](http://paperpile.com/b/RfW36U/5jdX)

37. Grkovic, T., Appleton, D. & Copp, B. Chemistry and chemical ecology of some of the common opisthobranch molluscs found on the shores of NE New Zealand. [*Chem. New Zealand,* **69**(4), 12 (2005).](http://paperpile.com/b/RfW36U/cryS)

38. [White-speckled Dorid (*Doriopsilla fulva*). *iNaturalist*](http://paperpile.com/b/RfW36U/3kyV) <https://www.inaturalist.org/taxa/467284-Doriopsilla-fulva>[.](http://paperpile.com/b/RfW36U/3kyV)

39. [Yellow-gilled Sea Goddess (*Doriopsilla gemela*). *iNaturalist*](http://paperpile.com/b/RfW36U/Zoyn) <https://www.inaturalist.org/taxa/479477-Doriopsilla-gemela>[.](http://paperpile.com/b/RfW36U/Zoyn)

40. Gibson, R. N., Atkinson, R. J. A. & Gordon, J. D. M. [*Oceanography and Marine Biology: An Annual Review, Volume 49*. (CRC Press, Boca Raton, FL, 2006). doi:](http://paperpile.com/b/RfW36U/CFv2)[10.1201/9781420006391](http://dx.doi.org/10.1201/9781420006391)[.](http://paperpile.com/b/RfW36U/CFv2)

41. Fahey, S. J. & Garson, M. J. Geographic variation of natural products of tropical nudibranch *Asteronotus cespitosus*. [*J. Chem. Ecol.* **28**, 1773–1785 (2002).](http://paperpile.com/b/RfW36U/gKjC)

42. Kubanek, J. & Andersen, R. J. Evidence for *de novo* biosynthesis of the polyketide fragment of diaulusterol A by the northeastern pacific dorid nudibranch *Diaulula sandiegensis.* [*J. Nat. Prod.* **62**, 777–779 (1999).](http://paperpile.com/b/RfW36U/sDdg)

43. Marin, A., Belluga, M. D. L., Scognamiglio, G. & Cimino, G. Morphological and chemical camouflage of the mediterranean nudibranch *Discodoris indecora* on the sponges *Ircinia variabilis* and *Ircinia fasciculata*. [*J. Molluscan Stud.* **63**, 431–439 (1997).](http://paperpile.com/b/RfW36U/SxrE)

44. Karuso, P. Chemical ecology of the nudibranchs. In [*Bioorganic Marine Chemistry* 31–60 (Springer Berlin Heidelberg, Berlin, Heidelberg, 1987).](http://paperpile.com/b/RfW36U/uaEp)

45. Fontana, A., Cavaliere, P., Wahidulla, S., Naik, C. G. & Cimino, G. A new antitumor isoquinoline alkaloid from the marine nudibranch Jorunna funebris. [*Tetrahedron* **56**, 7305–7308 (2000).](http://paperpile.com/b/RfW36U/xZwq)

46. Noble Dorid (*Peltodoris nobilis*). [*iNaturalist*](http://paperpile.com/b/RfW36U/wh7T) <https://www.inaturalist.org/taxa/48450-Peltodoris-nobilis>[.](http://paperpile.com/b/RfW36U/wh7T)

47. Penney, B. K. How specialized are the diets of Northeastern Pacific sponge-eating dorid nudibranchs? [*J. Molluscan Stud.* **79**, 64–73 (2013).](http://paperpile.com/b/RfW36U/AbGQ)

48. Gemballa, S. & Schermutzki, F. Cytotoxic haplosclerid sponges preferred: a field study on the diet of the dotted sea slug *Peltodoris atromaculata* (Doridoidea: Nudibranchia). [*Mar. Biol.* **144**, 1213–1222 (2004).](http://paperpile.com/b/RfW36U/319P)

49. Megina, C., Carballo, J. L., Cervera, J. L. & Garcia-Gomez, J. C. The diet of Platydoris Argo (Gastropoda: Nudibranchia) and the dietary specialization of sponge eating dorids. [*J. Molluscan Stud.* **68**, 173–179 (2002).](http://paperpile.com/b/RfW36U/ofaD)

50. Behrens, D. *Taringa aivica*. <https://slugsite.us/bow/nudwk329.htm> [(2002).](http://paperpile.com/b/RfW36U/efSm)

51. Duckworth, A. R. & Battershill, C. N. Population dynamics and chemical ecology of New Zealand Demospongiae *Latrunculia* sp. nov. and *Polymastia croceus* (Poecilosclerida: Latrunculiidae: Polymastiidae). [*N. Z. J. Mar. Freshwater Res.* **35**, 935–949 (2001).](http://paperpile.com/b/RfW36U/aePZ)

52. Goddard, J.H.R., 2007 (March 21) *Conualevia alba* Collier and Farmer, 1964 . *[In] Sea Slug Forum.* Australian Museum, Sydney. Available from http://www.seaslugforum.net/factsheet/conualba

53. [Hayward, P. J. & Ryland, J. S. *Handbook of the Marine Fauna of North-West Europe*. (Oxford University Press, London, England, 2017).](http://paperpile.com/b/RfW36U/Pyd4)

54. [Cimino, G. & Ghiselin, M. T. Chemical defense and evolutionary trends in biosynthetic capacity among dorid nudibranchs (Mollusca: Gastropoda: Opisthobranchia). *Chemoecology* **9**, 187–207 (1999).](http://paperpile.com/b/RfW36U/o0NL)

55. Gustafson, K. & Andersen, R. J. Chemical studies of British Columbia nudibranchs. [*Tetrahedron* **41**, 1101–1108 (1985).](http://paperpile.com/b/RfW36U/GDEk)

56. [Pawlik, J. R. Marine invertebrate chemical defenses. *Chem. Rev.* **93**, 1911–1922 (1993).](http://paperpile.com/b/RfW36U/9yaq)

57. [Wilson, N. G., Maschek, J. A. & Baker, B. J. A species flock driven by predation? Secondary metabolites support diversification of slugs in Antarctica. *PLoS One* **8**, e80277 (2013).](http://paperpile.com/b/RfW36U/BjpQ)

58. Rudman, W.B., 2000 (March 24) *Ancula gibbosa* (Risso, 1818). *[In] Sea Slug Forum.* Australian Museum, Sydney. Available from http://www.seaslugforum.net/factsheet/ancugibb

59. [Canning, M. H. & Carlton, J. T. Predation on kamptozoans (Entoprocta). *Invertebr. Biol.* **119**, 386–387 (2000).](http://paperpile.com/b/RfW36U/4Cxl)

60. [Parera, A. F. T., Pontes, M., Salvador, X. & Ballesteros, M. Sea-slugs (Mollusca, Gastropoda, Heterobranchia): the other inhabitants of the city of Barcelona (Spain). *Butlletí de la Institució Catalana d’Història Natural* 75–100 (2020).](http://paperpile.com/b/RfW36U/3Sio)

61. Goddard, J. The opisthobranchs of Cape Arago, Oregon, with notes on their natural history and a summary of benthic opisthobranchs known from Oregon [Master’s thesis, University of Oregon] (1984).

62. Rudman, W.B., 2001 (December 3) *Goniodoris nodosa* (Montagu, 1808). *[In] Sea Slug Forum.* Australian Museum, Sydney. Available from http://www.seaslugforum.net/factsheet/goninodo

63. [Edmunds, M. Acid secretion in some species of Doridacea (Mollusca, Nudibranchia). *J. Molluscan Stud.* **38**, 121–133 (1968).](http://paperpile.com/b/RfW36U/RtLC)

64. Parry, D. L. Chemical properties of the test of ascidians in relation to predation. *Mar. Ecol. Prog. Ser.* [**17**, 279–282 (1984).](http://paperpile.com/b/RfW36U/MRD6)

65. Robinson, N. Interactions Between The Nudibranch Okenia Zoobotryon And Its Bryozoan [Master’s thesis, University of Central Florida (2004).

66. Becerro, M. A., Starmer, J. A. & Paul, V. J. Chemical defenses of cryptic and aposematic Gastropterid molluscs feeding on their host sponge *Dysidea granulosa.* [*J. Chem. Ecol.* **32**, 1491–1500 (2006).](http://paperpile.com/b/RfW36U/FPOt)

67. *Trapania reticulata*. [*iNaturalist*](http://paperpile.com/b/RfW36U/ShXr) <https://www.inaturalist.org/taxa/949643-Trapania-reticulata>[.](http://paperpile.com/b/RfW36U/ShXr)

68. [Valdés, À. & Gosliner, T. M. Phylogeny of the radula‐less dorids (Mollusca, Nudibranchia), with the description of a new genus and a new family. *Zool. Scr.* **28**, 315–360 (2002).](http://paperpile.com/b/RfW36U/IFvB)

69. [Mikhlina, A. L., Tzetlin, A. B., Ekimova, I. A. & Vortsepneva, E. V. Drilling in the dorid species *Vayssierea cf. elegans* (Gastropoda: Nudibranchia): Functional and comparative morphological aspects. *J. Morphol.* **280**, 119–132 (2019).](http://paperpile.com/b/RfW36U/DjyK)

70. Rudman, W.B., 2005 (November 10) *Acanthodoris nanaimoensis* O'Donoghue 1921. *[In] Sea Slug Forum.* Australian Museum, Sydney. Available from http://www.seaslugforum.net/find/acannana

71. Graziani, E. I. & Andersen, R. J. Investigations of sesquiterpenoid biosynthesis by the dorid nudibranch *Acanthodoris nanaimoensis*. [*J. Am. Chem. Soc.* **118**, 47014701 (1996).](http://paperpile.com/b/RfW36U/s8sn)

72. White, H. Relationships between the nudibranch *Adalaria proxima* and its prey, the bryozoan *Electra pilosa* [Doctoral thesis, University of St. Andrews] (1993).

73. Martynov, A. & Schrödl, M. Phylogeny and evolution of corambid nudibranchs (Mollusca: Gastropoda). [*Zool. J. Linn. Soc.* **163**, 585–604 (2011).](http://paperpile.com/b/RfW36U/V9IY)

74. [Furfaro, G. *et al.* Mediterranean matters: Revision of the family Onchidorididae (Mollusca, Nudibranchia) with the description of a new genus and a new species. *Diversity (Basel)* **15**, 38 (2023).](http://paperpile.com/b/RfW36U/5Ls7)

75. [Havenhand, J. N. & Todd, C. D. Physiological ecology of *Adalaria proxima* (Alder et Hancock) and *Onchidoris muricata* (Müller) (Gastropoda: Nudibranchia): Feeding, growth, and respiration. *J. Exp. Mar. Bio. Ecol.* **118**, 151–172 (1988).](http://paperpile.com/b/RfW36U/UpWp)

76. Iyengar, E. V. & Harvell, C. D. Specificity of cues inducing defensive spines in the bryozoan *Membranipora membranacea*. [*Mar. Ecol. Prog. Ser.* **225**, 205–218 (2002).](http://paperpile.com/b/RfW36U/7Ox9)

77. Rudman, W.B., 2001 (Jul 5). Comment on *Onchidoris muricata* from New England by Paul Young. *[Message in] Sea Slug Forum.* Australian Museum, Sydney. Available from http://www.seaslugforum.net/find/4712

78. [Chichvarkhin, A., Chichvarkhina, O., Ekimova, I. & Chalenko, K. First record of nudibranch mollusk *Onchidoris muricata* (O. F. Müller, 1776) (Mollusca, Gastropoda, Heterobranchia) in the Sea of Japan and its ephemeral population associated with unusual prey. *Mar. Biodivers.* **48**, 1571–1578 (2016).](http://paperpile.com/b/RfW36U/NSjd)

79. [Wu, Q. *et al.*](http://paperpile.com/b/RfW36U/Lp2w) Cytotoxic nitrogenous terpenoids from two South China Sea nudibranchs *Phyllidiella pustulosa*, *Phyllidia coelestis*, and their sponge prey *Acanthella cavernosa*. [*Mar. Drugs* **17**, 56 (2019).](http://paperpile.com/b/RfW36U/Lp2w)

80. [Fusetani, N. *et al.*](http://paperpile.com/b/RfW36U/In8h) Two sesquiterpene isocyanides and a sesquiterpene thiocyanate from the marine sponge *Acanthella cf. cavernosa* and the Nudibranch *Phyllidia ocellata*. [*Tetrahedron Lett.* **33**, 6823–6826 (1992).](http://paperpile.com/b/RfW36U/In8h)

81. Yasman, Y., Edrada, R. A., Wray, V. & Proksch, P. New 9-thiocyanatopupukeanane sesquiterpenes from the nudibranch *Phyllidia varicosa* and its sponge-prey *Axinyssa aculeata*. [*J. Nat. Prod.* **66**, 1512–1514 (2003).](http://paperpile.com/b/RfW36U/t3B9)

82. Dumdei, E. J., Flowers, A. E., Garson, M. J. & Moore, C. J. The biosynthesis of sesquiterpene isocyanides and isothiocyanates in the marine sponge *Acanthella cavernosa*; Evidence for dietary transfer to the dorid nudibranch *Phyllidiella pustulosa.* [*Comp. Biochem. Physiol. A Comp. Physiol.* **118**, 1385–1392 (1997).](http://paperpile.com/b/RfW36U/wUC4)

83. [Jaisamut, S. *et al.*](http://paperpile.com/b/RfW36U/8lBs) Bridged tricyclic sesquiterpenes from the tubercle nudibranch *Phyllidia coelestis* Bergh. [*J. Nat. Prod.* **76**, 2158–2161 (2013).](http://paperpile.com/b/RfW36U/8lBs)

84. van Alphen, J., de Voogd, N. J. & Hoeksema, B. W. Differential feeding strategies in phyllidiid nudibranchs on coral reefs at Halmahera, northern Moluccas. [*Coral Reefs* **30**, 59–59 (2011).](http://paperpile.com/b/RfW36U/xd1I)

85. [Manzo, E. *et al.*](http://paperpile.com/b/RfW36U/vBT4) Isocyanide terpene metabolites of *Phyllidiella pustulosa*, a nudibranch from the South China Sea. [*J. Nat. Prod.* **67**, 1701–1704 (2004).](http://paperpile.com/b/RfW36U/vBT4)

86. Lyakhova, E. G., Kolesnikova, S. A., Kalinovskii, A. I. & Stonik, V. A. Secondary metabolites of the Vietnamese nudibranch mollusk *Phyllidiella pustulosa*. [*Chem. Nat. Compd.* **46**, 534–538 (2010).](http://paperpile.com/b/RfW36U/W6tq)

87. Wright, A. D. GC-MS and NMR analysis of *Phyllidiella pustulosa* and one of its dietary sources, the sponge *Phakellia carduus*. [*Comp. Biochem. Physiol. A Mol. Integr. Physiol.* **134**, 307–313 (2003).](http://paperpile.com/b/RfW36U/mPjm)

88. Rudman, W.B., 2007 (Apr 16). Comment on *Reticulidia halgerda* from the Philippines by Mike Krampf. *[Message in] Sea Slug Forum.* Australian Museum, Sydney. Available from http://www.seaslugforum.net/find/19568

89. Geange, S. W. & Stier, A. C. Charismatic microfauna alter cyanobacterial production through a trophic cascade. [*Coral Reefs* **29**, 393–397 (2010).](http://paperpile.com/b/RfW36U/kbCI)

90. Nakano, R., Tanaka K. O. T. A. R. O., Dewa, S., Takasaki, K. & Ono, A. Field observations on the feeding of the nudibranch *Gymnodoris* spp. in Japan. [*Veliger* **49**, 91–96 (2007).](http://paperpile.com/b/RfW36U/J6v0)

91. Nakano, R. & Hirose, E. Field Experiments on the feeding of the nudibranch *Gymnodoris* spp. (Nudibranchia: Doridina: Gymnodorididae) in Japan. [*Veliger* **51**, 66–75 (2011).](http://paperpile.com/b/RfW36U/l1Xt)

92. Nakano, R., Uochi, J., Fujita, T. & Hirose, E. *Kalinga ornata* Alder & Hancock, 1864 (Nudibranchia: Polyceridae): a unique case of a sea slug feeding on echinoderms. [*J. Molluscan Stud.* **77**, 413–416 (2011).](http://paperpile.com/b/RfW36U/jZU2)

93. Nakano, R. Natural feeding habits of two nudibranchs: [*Kalinga ornata* and *Plocamopherus tilesii* (Nudibranchia: Doridacea: Polyceridae). *Molluscan Res.* **37**, 153–157 (2017).](http://paperpile.com/b/RfW36U/qwTX)

94. Selvam, D., Gunasekaran, K. & Saravanakumar, A. Food and feeding biology of Kalinga ornata (alder & Hancock, 1864) from Pazhayar, southeast coast of India. [*IJSIT* (2016).](http://paperpile.com/b/RfW36U/yoUw)

95. Rudman, W.B., 2007 (May 18) *Lecithophorus capensis* Macnae, 1958. *[In] Sea Slug Forum.* Australian Museum, Sydney. Available from http://www.seaslugforum.net/factsheet/lecicape

96. Uribe, R. A., Sepúlveda, F., Goddard, J. H. R. & Valdés, Á. Integrative systematics of the genus *Limacia* O. F. Müller, 1781 (Gastropoda, Heterobranchia, Nudibranchia, Polyceridae) in the Eastern Pacific. [*Mar. Biodivers.* **48**, 1815–1832 (2018).](http://paperpile.com/b/RfW36U/2cGd)

97. Caballer Gutiérrez, M., Almón, B. & Pérez-Dieste, J. The sea slug genus *Limacia* Müller, 1781 (Mollusca: Gastropoda: Heterobranchia) in Europe. [*Sede Central IEO* (2016).](http://paperpile.com/b/RfW36U/srB3)

98. Graziani, E. I. & Andersen, R. J. Limaciamine, a new Diacylguanidine Isolated from the North Sea nudibranch *Limacia clavigera*. [*J. Nat. Prod.* **61**, 285–286 (1998).](http://paperpile.com/b/RfW36U/lizO)

99. Rudman, W.B., 2007 (Aug 8). Comment on *Nembrotha cristata* feeding by Dr. Matt Doggett. *[Message in] Sea Slug Forum.* Australian Museum, Sydney. Available from http://www.seaslugforum.net/find/20385

100. Paul, V. J., Lindquist, N. & Fenical, W. Chemical defenses of the tropical ascidian *Atapozoa* sp. and its nudibranch predators *Nembrotha* spp. [*Mar. Ecol. Prog. Ser.* **59**, 109–118 (1990).](http://paperpile.com/b/RfW36U/CJmH)

101. [Hamel, J.-F., Sargent, P. & Mercier, A. Diet, reproduction, settlement and growth of *Palio dubia*](http://paperpile.com/b/RfW36U/6bL9) (Nudibranchia: Polyceridae) in the north-west Atlantic. [*J. Mar. Biol. Assoc. U. K.* **88**, 365–374 (2008).](http://paperpile.com/b/RfW36U/6bL9)

102. [Carbone, M. *et al.* Occurrence of symmetrical diacylguanidines triophamine and limaciamine in three Polyceridae species from Canary Islands: are they chemical markers of these nudibranchs? *Biochem. Syst. Ecol.* **83**, 62–65 (2019).](http://paperpile.com/b/RfW36U/OqbS)

103. [Sørensen, C. G., Rauch, C., Pola, M. & Malaquias, M. A. E. Integrative taxonomy reveals a cryptic species of the nudibranch genus *Polycera*](http://paperpile.com/b/RfW36U/qFl3) (Polyceridae) in European waters. [*J. Mar. Biol. Assoc. U. K.* **100**, 733–752 (2020).](http://paperpile.com/b/RfW36U/qFl3)

104. [Davidson, S. K. Biology of the bryostatins in the marine bryozoan *Bugula* *neritina*: Symbiosis, cryptic speciation and chemical defense [Doctoral thesis, University of California San Diego] (1999).](http://paperpile.com/b/RfW36U/YfXv)

105. [Lim, G. E. L. D. *Bugula* (Bryozoa) and their bacterial symbionts: A study in symbiosis, molecular phylogenetics and secondary metabolism [Doctoral thesis, University of California San Diego] (2004).](http://paperpile.com/b/RfW36U/4UxG)

106. [Willan, R. C. & Chang, Y.-W. Description of three new species of Tambja (Gastropoda, Nudibranchia, Polyceridae) from the western Pacific Ocean reveals morphological characters with taxonomic and phylogenetic significance for tradictional Polyceridae and related ‘phaneorobranch’ nudibranchs. *Basteria* **81**, 1–23 (2017).](http://paperpile.com/b/RfW36U/C746)

107. [Pola, M., Padula, V., Gosliner, T. M. & Cervera, J. L. Going further on an intricate and challenging group of nudibranchs: description of five novel species and a more complete molecular phylogeny of the subfamily Nembrothinae (Polyceridae). *Cladistics* **30**, 607–634 (2014).](http://paperpile.com/b/RfW36U/SWMe)

108. [Carté, B. & Faulkner, D. J. Role of secondary metabolites in feeding associations between a predatory nudibranch, two grazing nudibranchs, and a bryozoan. *J. Chem. Ecol.* **12**, 795–804 (1986).](http://paperpile.com/b/RfW36U/6RGZ)

109. Faulkner, D. J., Molinski, T. F., Andersen, R. J., Dumdei, E. J. & De Silva, E. D. Geographical variation in defensive chemicals from Pacific coast dorid nudibranchs and some related marine molluscs. [*Comp. Biochem. Physiol. C* **97**, 233–240 (1990).](http://paperpile.com/b/RfW36U/YbTM)

110. Behrens, D. *Goniodoridella* sp. https://slugsite.us/bow2007/nudwk886.htm (2014).

111. Thompson, T.E. Molluscs: Benthic opisthobranchs (Mollusca: Gastropoda). synopses of the British fauna (New Series). 2nd Edition. Leiden. 1–356 (1988).

112. Paz-Sedano, S., Moles, J., Smirnoff, D., Gosliner, T. M. & Pola, M. A combined phylogenetic strategy illuminates the evolution of Goniodorididae nudibranchs (Mollusca, Gastropoda, Heterobranchia). *Mol. Phylo. Evol.* **192**, 107990 (2024).
